# Rumen (*Calicophoron*/*Paramphistomum* spp.) and Liver Flukes (*Fasciola hepatica*) in Cattle—Prevalence, Distribution, and Impact of Management Factors in Germany

**DOI:** 10.3390/ani11092727

**Published:** 2021-09-18

**Authors:** Tanja Forstmaier, Gabriela Knubben-Schweizer, Christina Strube, Yury Zablotski, Christoph Wenzel

**Affiliations:** 1 Clinic for Ruminants with Ambulatory and Herd Health Services, Centre for Clinical Veterinary Medicine, Ludwig-Maximilians-Universität München, 85764 Oberschleissheim, Germany; T.Forstmaier@med.vetmed.uni-muenchen.de (T.F.); gknubben@med.vetmed.uni-muenchen.de (G.K.-S.); Y.Zablotski@med.vetmed.uni-muenchen.de (Y.Z.); 2 Institute for Parasitology, Centre for Infection Medicine, University of Veterinary Medicine Hannover, 30559 Hanover, Germany; Christina.Strube@tiho-hannover.de

**Keywords:** paramphistomidosis, rumen flukes, *Calicophoron daubneyi*, *Paramphistomum leydeni*, coinfection, faecal examination, modelling, risk factors, organic farming, ruminants

## Abstract

**Simple Summary:**

Paramphistomidosis is a parasitic disease of ruminants caused by so-called rumen flukes. To determine the current prevalence in German cattle, faecal samples from 614 herds were examined for parasite eggs. In addition, the occurring rumen fluke species were determined, resulting in *Calicophoron daubneyi* and *Paramphistomum leydeni*. In the course of the work, the occurrence of the common liver fluke, causing fasciolosis, was also documented. Rumen fluke eggs could be detected in 5.5% of German cattle farms, liver flukes in 9.5%. In 2.1% of the farms, both fluke types occurred. Regional differences between northern and southern Germany were detected. Cattle grazing and fed with fresh grass were more likely to be infected with rumen and liver flukes than cattle without such access. Cattle from organic farms were also more likely to have fluke infections than those from conventional farms, in suckler cows, however, this association only applied to rumen flukes.

**Abstract:**

This study was carried out to determine the prevalence of rumen flukes on German cattle farms via the sedimentation technique, and to identify the rumen fluke species occurring in Germany. Additionally, the prevalence of patent *Fasciola hepatica* infections was determined. Furthermore, a short questionnaire was answered by the farmers. A prevalence of 5.5% and 9.5% was detected for rumen flukes and liver flukes, respectively. Coinfections occurred on 2.1% of farms. In northern Germany, the rumen fluke prevalence was higher than in southern Germany, while for liver fluke the distribution was reversed. Rumen flukes were mostly identified as *Calicophoron daubneyi*, but in four cases, sequencing revealed *Paramphistomum leydeni* for the first time in Germany. Grazing and feeding of fresh grass, as well as organic farming, were significantly associated with rumen and liver fluke occurrence. In contrast, suckler cow husbandry only had an influence on the occurrence of rumen flukes, but not liver flukes. Trematode eggs could be detected in both, farms with and without deworming. Since there were only a few studies about Paramphistomidosis in Germany, more attention should be paid to these parasitic diseases for animal welfare and animal health reasons.

## 1. Introduction

Trematodes of the family Paramphistomidae, so-called rumen flukes, e.g., *Paramphistomum cervi*, *Paramphistomum leydeni,* or *Calicophoron daubneyi*, cause paramphistomidosis in ruminants [1]. Rumen flukes mainly infect domestic and wild ruminants [2], but also new world camelids may be affected [3]. Parasite development includes an intermediate host, represented by different genera of air-breathing freshwater snails [4]. *Calicophoron daubneyi*, nowadays the most frequently recorded rumen fluke in northern, southern, and western Europe [5], and the liver fluke *Fasciola hepatica* share the same intermediate host, the dwarf pond snail *Galba truncatula* [6]. Therefore, coinfections are possible in both the intermediate, as well as the final host [7,8]. Infection of the final host with rumen fluke metacercariae is followed by two phases. During the intestinal phase, juvenile flukes migrate within the mucosa of the small intestine and abomasum towards the rumen. This may result in diarrhoea and weight loss up to cachexia, and, in case of massive infection, even death of the animals [9,10,11]. In the ruminal phase, flukes attach to the mucosa and mature into adult egg-laying worms. Ruminal paramphistomidosis is usually clinically inapparent, despite the occurrence of pathological lesions caused by the parasites [12].

Currently, coproscopy by sedimentation is the only practicable method for diagnosing rumen fluke infections in live animals, although it can only detect patent infections [13]. During prepatency, faeces can be sieved, and the material retained examined for juvenile stages with a stereomicroscope; however, the method is only meaningful in positive cases. *Post mortem* detection includes the examination of the upper digestive tract at the abattoir or during necropsy. Serological methods are not commercially available yet. Recently, however, a coproantigen-based ELISA with high analytical sensitivity and specificity has been developed [14], which is a promising future tool to detect current infections in routine diagnosis or epidemiological studies.

A few years ago, paramphistomidosis has been reported as an emerging parasitic disease in ruminants in Europe [15]. For cattle, prevalences of up to 45% positive herds in central France [16], up to 32% in the retrospective analysis of laboratory records [17] and 52% at abattoirs in Ireland [18], 59% positive herds in Wales [19], 61% positive herds [20], and 6% and 19% at abattoirs in north-western Spain [21,22], 22% positive herds and 28% at abattoirs in Belgium [23], and 16% positive herds in the Netherlands [24] have been determined. So far, no comparable figures are available for cattle in Germany. From 1950 to 2000, only a few case reports have been published [25]. Based on recent findings of *C. daubneyi* in German cattle herds, it can be assumed that the parasitosis is also spreading in this country [26]. For the liver fluke *Fasciola hepatica*, a seroprevalence of 15% was most recently reported for Germany, and the risk of infection was associated, among other factors, with grazing and feeding fresh grass [27,28]. Due to the same intermediate host, on all farms with liver fluke infection, cattle can potentially also get infected with *C. daubneyi*. For example, on 46% of Welsh farms coinfections of these two trematodes were observed [19].

Therefore, one aim of this study was to determine the prevalence of paramphistomidosis in German cattle herds and to identify the infecting rumen fluke species. In order to present comprehensive data on fluke infections and to take into account the shared intermediate host *G. truncatula*, the study also aimed to investigate the prevalence of patent *F. hepatica* infections, and to determine risk factors for both trematodes.

## 2. Materials and Methods

### 2.1. Sample Size Calculation and Sampled Farms

At the beginning of the study, the sample size was calculated to collect enough samples for reliable statistical analysis. A rather low prevalence of 5.0% positive herds was assumed, as there are only few reports for Germany so far [25,26]. Additionally, the seroprevalence of 17.7% for *F. hepatica* in the federal state of Bavaria [27] was included. Faecal samples were collected from October 2018 to December 2020. Sampling was carried out as part of the German research project “PraeRi” [29] in dairy farms (*n* = 285), and via media acquisition (calls in different journals and social media) as well as direct contact to veterinary practices, animal health funds and animal health services (*n* = 329). In total, 614 German farms were sampled. To take account of regional differences, Germany was divided into four regions. In the region North, 179 farms were sampled, in East 76 farms, in South 277 farms and in West 82 farms (Table 1). Resulting prevalences were calculated for Germany as a whole, each of the four regions and, additionally, the federal states of Bavaria (*n* = 205) and Lower Saxony (*n* = 92), because these federal states include the highest number of cattle in Germany and the required sample size has been achieved. Farms with co-infections were included in the calculations for both rumen flukes and common liver fluke, respectively.

### 2.2. Questionnaire Survey

The questionnaire provided to the participating farmers included the following questions: federal state, cattle breed, production type (dairy cattle/fattening beef cattle/suckler beef cattle farming [hereafter referred to as suckler cow]), agricultural system (organic/conventional), grazing patterns or feeding of fresh grass, number of young and adult animals, deworming scheme and known problems with rumen or liver flukes. Faecal sampling was carried out according to a provided manual, either during defecation or by collecting freshly excreted faeces. The sampling scheme included eight young (six months to 2.5 years or before first calving) and eight adult cattle (older than 2.5 years or after first calving) at each farm, and samples of four animals each were pooled for coproscopical analyses. Overall, 90% of farms complied with this pattern (*n* = 555), but the other 59 farms (less samples collected than specified and only from young or adult cattle) were also included in the final analysis.

### 2.3. Coproscopical Examination

Samples received were refrigerated for one day up to eight weeks prior to examination with the sedimentation technique, which was carried out as follows: First, the pooled sample was thoroughly mixed and approximately 10 g faeces were homogenised with tap water. The obtained homogenous suspension was washed through a sieve (mesh size–1500 µm) into a 600 mL beaker by rinsing the sieve with a strong water jet until the beaker was filled to the 500 mL mark. Each sample was allowed to sediment for at least 15 min before the supernatant was decanted and the beaker refilled with tap water. This process was repeated until the supernatant became clear. The received sediment was transferred into a petri dish through a fine-meshed sieve (mesh size 300 µm). Approximately 8 mL of the sediment were pipetted in an examining chamber, where the sample was stained with three drops 1% methylene blue and examined microscopically for fluke eggs, which were differentiated by their colour.

### 2.4. Molecular Identification of Rumen Fluke Species

From samples positive for rumen flukes, usually 10–20 eggs were isolated and subjected to species identification by amplifying and sequencing the ITS-2 region, as described previously [26]. In brief, genomic DNA was extracted from the eggs with the DirectPCR^®^ Lysis Reagent (Cell) (PEQLAB Biotechnologie GmbH, Erlangen, Germany) and amplified in a 50 µL reaction volume containing 1 µL DreamTaq DNA Polymerase (5 U/µL) (ThermoFisher Scientific, Schwerte, Germany), 5 µL 10x DreamTaq buffer, 1 µL dNTP mix (10 mM each), 2 µL of each primer (ITS-2For and ITS-2Rev [30], 10 µM each), and 10 µL DNA template. Thermocycling was conducted at 95 °C for 3 min, 40 cycles of 95 °C for 30 s, 53 °C for 1 min, 72 °C for 45 s, and 72 °C for 10 min. The amplification products were custom-sequenced (Seq-lab Sequence Laboratories, Göttingen, Germany) and compared with available sequences in NCBI (National Center for Biotechnology Information) GenBank.

### 2.5. Statistical Analysis

The statistical analyses were carried out using R version 4.0.3. (The R Foundation for Statistical Computing, Vienna, Austria) [31] and Microsoft Excel 2019 (Microsoft Corporation, Redmond, WA, USA). The 95% confidence interval (CI) for rumen and liver fluke prevalences in Germany were calculated using Wald approximation. Logistic regression was carried out to estimate prevalences in the four study regions and two federal states (Bavaria and Lower Saxony), as well as for the production type (dairy/suckler cows) and the agricultural system (organic/conventional). All contrasts (differences) were assessed after model-fitting by the estimated least-squares marginal means (emmeans) with the Tukey *p*-value correction for multiple comparisons [32]. The first level of any variable was used as an intercept. Associations of the variable “coproscopic result” with “agricultural system”, “breed”, and “grazing/feeding fresh grass” were tested via a Pearson’s Chi-Square Test. *p*-values ≤ 0.05 were considered statistically significant in all analyses.

## 3. Results

### 3.1. Study Population

Of the 614 participating farms, 571 were dairy farms (region North: *n* = 173, East: *n* = 57, South: *n* = 270, West: *n* = 71) and 43 suckler cow farms (North: *n* = 6, East: *n* = 19, South: *n* = 7, West: *n* = 11). No fattening beef farms participated in the study. In total, 506 conventional and 106 organic farms took part in the study, two farms did not provide information. On 457 farms, cattle where grazed or fed with fresh grass, while on 156 farms this was not the case. One farmer did not provide any information on grazing and feeding fresh grass at all.

### 3.2. Prevalence of Rumen and Liver Flukes in Germany

**Figure 1:**
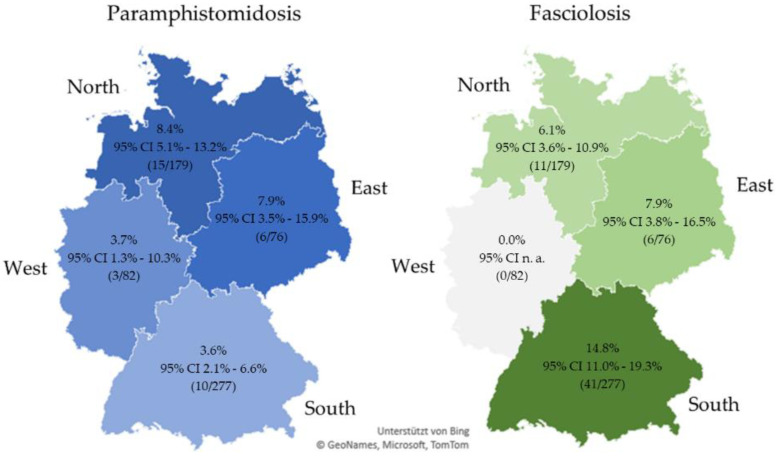
Prevalence and 95% confidence interval for rumen fluke and *F. hepatica* prevalence in German regions (coinfections included). Abbreviations: CI, confidence interval; n. a., not applicable.

The rumen fluke prevalence in Germany amounted to 5.5% (95% CI 3.7–7.4%, 34/614). The highest prevalence was observed in the region North (8.4%), the lowest in South (3.6%). For *F. hepatica*, a prevalence of 9.5% (95% CI 7.1–11.8%, 58/614) was determined for Germany. The region South revealed the highest prevalence with 14.8%, whereas in West no farm was tested positive. Detailed regional results are provided in Figure 1. Coinfections were identified on 13 German farms (2.1%, 95% CI 1.0–3.3%). The highest regional coinfection rate was determined in East with coinfections on four farms (5.3%, 95% CI 1.9–12.4%), while logically no coinfections occurred in West (0.0%, 95% CI n. a.). In North, coinfections were detected on three farms (1.7%, 95% CI 0.6–4.9%) and in South on six farms (2.2%, 95% CI 0.1–4.5%).

In the statistical comparison of the regions, only the rumen fluke prevalence difference between the regions North and South was statistically significant. Farms in North have a 2.3 time higher odds of their cattle being infected than farms in South (Table 2). These regional differences were also reflected when comparing the two federal states with the highest number of cattle in Germany: In the federal state Lower Saxony (North), rumen fluke prevalence was 10.9% (10/92, 95% CI: 5.8%–18.6%) compared to 4.4% in Bavaria (South; 9/205, 95% CI 2.4%–8.3%), decreasing the odds for a positive result in Bavaria by 0.4 (*p* = 0.047).

The comparison between North and South was also statistically significant for *F. hepatica*. Cattle on a farm in North have 0.4 times lower odds of being infected with liver flukes than those in South (Table 2). Again, comparison of the two federal states mirrored this picture: Liver fluke prevalence in Lower Saxony was 6.5% (6/92, 95% CI: 3.2%–13.9%) and in Bavaria 16.1% (33/205, 95%CI: 11.6%–21.6%), where a farm has 2.6 times higher odds of its cattle being infected than a farm in Lower Saxony (*p* = 0.031).

### 3.3. Rumen Fluke Species Identification

Molecular identification of infecting rumen fluke species was successful in 24 of the 34 affected farms. *Calicophoron daubneyi* was identified on 20 farms, seven of which were located in the region North, five each in East and South, and three in West. *Paramphistomum leydeni* was detected in the four remaining farms, three of them in North and one in East. Five samples each from the regions North and South could not be identified.

### 3.4. Impact of Management Factors on Rumen and Liver Fluke Occurrence and Awareness of Farmers

The frequency of patent rumen and liver fluke infections in relation to the management factors production type and agricultural system is shown in Table 3. Due to a rather inhomogeneous and partly incomplete data, the management factors grazing/feeding fresh grass and anthelmintic treatment were only evaluated descriptively (Table 4).

Rumen fluke prevalence in German dairy herds was 4.0% (95% CI: 2.8%–6.1%) and in suckler cow herds 25.6% (95% CI: 14.2%–39.4%). According to these results, suckler cow herds have 7.7 times higher odds of being infected with rumen flukes than dairy herds (*p* < 0.001). *Fasciola hepatica* prevalence in dairy herds amounted to 9.5% (95% CI: 7.3%–12.1%) and in suckler cow herds to 9.3% (95% CI: 3.7%–21.6%) with no statistically significant difference between these production types (*p* = 0.974). Cattle breeds on dairy farms were predominantly Holstein Friesian in the North, East, and West, and German Simmental or German Brown Swiss in the South. There was no statistical correlation between the breed and rumen fluke infections (*p* = 0.07). In contrast, a significant correlation was found between the breed and *F. hepatica* infections, since this fluke species was detected more often on farms with German Simmental herds compared to farms with Holstein Friesian herds (*p* < 0.001). Overall, on the farms tested positive for flukes, mainly adult animals were affected.

Both rumen and liver fluke infections showed a statistically significant relationship with organic agriculture, where cattle were more often infected than on conventional farms (*p* = 0.01 and *p* < 0.001, respectively). Regarding diet, grazing was evaluated together with feeding of fresh grass. Cattle grazing or fed with fresh grass were more likely to be infected with rumen and liver fluke than cattle without such access (*p* < 0.001). Notably, three positive dairy farms (rumen flukes once, liver fluke twice) categorised as “not grazing” did not provide information about feeding of fresh grass.

Deworming was carried out on 274 farms, not on 338 farms, and two farms did not provide any information. Of the farms that treat with anthelmintics, 29 use fasciolicides (10 use triclabendazole, 7 oxyclozanide, 6 closantel, 4 albendazole, 1 oxyclozanide + levamisole + triclabendazole, 1 oxyclozanide + closantel). Of these, 23 farms had dairy cows and six had suckler cows. In relation to the total number of these production types, more suckler cow herds are treated with fasciolicides than dairy cow herds. None of the farms without access to grazing or fresh grass reported to deworm with faciolicides. A number of 101 farms dewormed with other drugs, mostly those against gastrointestinal nematodes. No information on the drug used was provided by 144 farms.

## 4. Discussion

The expected rumen fluke prevalence of 5.0% in Germany is in line with the prevalence of 5.5% determined by this study. This is a low figure compared to other European studies reporting prevalences between 6% and 61% positive herds in the studied region or country [16,17,18,19,20,21,22,23,24]. However, the comparability with other studies is not always reliable due to different approaches. In some studies, samples were taken only at one abattoir [23], or data from veterinary laboratories were evaluated retrospectively [16,17,18]. Furthermore, prevalences differ between individual animal or herd level [20,24]. In the present study, representativity is probably given by reaching the predefined number of samples in every region. However, only the samples collected via the PraeRi project [29] were randomly selected, while the other farms were based on the farmers’ willingness to participate in the call, a compromise that had to be made to ensure high numbers of farms within the four regions. The questionnaire was designed to provide the most objective answers possible. On 285 farms, trained and coordinated interviewers using a standard procedure filled the questionnaire, in the other cases the farmers did it themselves.

Paramphistomidosis was detected throughout Germany, as rumen fluke eggs were found in farms of all four regions. This finding is in line with Huson et al. [15], who identified paramphistomidosis as an emerging parasitic disease in Europe. Interestingly, prevalences were significantly higher in the north than the south of Germany, while the opposite was true for *F. hepatica* infections. This was unexpected since both trematodes share the intermediate host *G. truncatula* and, thus, have a similar epidemiology [33,34]. One reason for the contrariness between north and south might be a competition of rumen and liver fluke stages within the intermediate host. This is supported by Jones et al. [19], who found a significant negative correlation between *F. hepatica* and *C. daubneyi* infection intensities in farms in the UK. Another possible reason is, that Bavaria due to the typical local breed (German Simmental) has probably less international animal trade than other regions with worldwide more “common” breeds such as Holstein Friesian. If this is true, then we expect a slow increase in rumen flukes in south Germany going more in line with liver fluke prevalence in the future. Nevertheless, this result of our study requires further research.

In eastern Germany, the same prevalence values were determined for rumen and liver flukes, with the latter occurring significantly less frequently than in the south. Surprisingly, no liver fluke eggs were detected in samples from the west (exclusively based on the farmers’ willingness to participate), although patent infections in cattle were recently reported [26]. One possible explanation is that the sample size (86 samples) from this region was too small to detect a possibly very low *F. hepatica* infection extensity, or that farmers may have chosen not to participate if they had known problems with fasciolosis. Unfortunately, no other recent data on the occurrence on liver flukes in western Germany are available. In a bulk tank milk (BTM) study conducted in 2008, seroprevalences amounted to 9–19% in the German federal states included in the region “West” [27], but this region was not included in a recent BTM study on fasciolosis in Germany examining samples from 2017 to 2019 [28]. Although seroprevalence values are difficult to compare with the prevalence determined in our investigation, as the sensitivity differs and only patent infections can be detected by coproscopy [13,35], the most recent seroprevalence study confirms higher exposure to liver flukes in the south than in the north of Germany [28]. However, *F. hepatica* BTM seroprevalence in the region “East” was only 1% [28] compared to nearly 8% patent infections in our study. This may be explained by different sample sets (BTM samples were collected in 2017–2019, faecal samples in 2018–2020, and participating farms overlapped only partially), but needs to be clarified in future studies.

Overall, the reason for the observed regional differences in patent rumen and liver fluke infections cannot be reliably explained by this study. Other authors assume climatic and environmental factors, as well as the import of animals as possible reasons for regional differences in prevalence [19,36,37]. Again, further research is required to elucidate underlying epidemiological factors.

The detection of *C. daubneyi* in all four regions is in line with the findings at European level, as this species has been mainly detected in recent decades [16,17,19,22,24]. In contrast, *P. leydeni*, which occurred on four farms, was identified for the first time in Germany. In Europe, *P. leydeni* has so far been found rarely overall [24,36,38], but in Argentina it appears to be the most ubiquitous species [39]. In contrast to *C. daubneyi*, whose intermediate host is the lymnaeid snail *G. truncatula*, *P. leydeni* has planorbid snails as intermediate hosts. However, in order to be able to analyse the distribution pattern of the two rumen fluke species more precisely, the epidemiology of the various Paramphistomidae must be further investigated.

Cattle grazing or fed with fresh grass were more likely to be infected with rumen or liver fluke than cattle without such access. This result was expected since the development of the parasites requires freshwater snails as intermediate host and infective metacercariae encyst on plants. The variable “grazing/ feeding fresh grass” is very likely the reason for the higher prevalence of both fluke types on organic farms, because due to the EU legislation cattle must have permanent access to open space, preferably to pastures. Additionally, grazing and feeding of fresh grass seems to be the reason for the higher risk of suckler cows of being infected with rumen flukes compared to dairy cows. This result is consistent with studies from Spain and the Netherlands [22,24]. In our study, a very similar prevalence of *F. hepatica* in dairy and suckler cow farms was found. The reason for this could be that suckler cow herds are more likely to be dewormed with fasciolicides, as they are known to have a higher risk of infection compared to dairy herds. Since treatment with triclabendazole and closantel against liver flukes is usually not effective against rumen fluke infection [5], the prevalence of rumen flukes remains high. In addition, when ruminants are treated against *F. hepatica*, egg excretion on pastures is reduced. As a result, rumen flukes have less competition at the intermediate host level and are, therefore, able to infect more snails [19].

An unexpected result is the detection of trematode infections in three farms without grazing (rumen flukes once, liver fluke twice) in this study. As there was no information on the feeding regime available, it can only be assumed that the feeding of fresh grass or field dried hay is the cause of the infection. The possibility of infection by feeding fresh grass or field dried hay contaminated with metacercariae has already been proved for *F. hepatica* [40,41] and this could also apply to an infection with rumen flukes [21]. It would also be possible that cattle already infected elsewhere entered the farm via livestock trades. This has already been considered by other authors as a cause for the detection of trematode infections in farms without grazing [17,21].

The evaluation of the susceptibility of different cattle breeds to rumen and liver flukes cannot be conclusively assessed due to insufficient representativity of the samples. In dairy farms, it was not possible to establish a link between rumen fluke detection and the cattle breed tested. This is in line with other published results [21,37,42]. The breed distribution in suckler cow farms was too various for analysis. Although German Simmental cattle were more frequently infected with *F. hepatica* than Holstein Friesians, this is most likely due to the generally higher liver fluke prevalence in the south of Germany, where mainly German Simmental cattle are kept. For this reason, a predisposition of a specific cattle breed is rather unlikely. In most of the farms studied, rumen and liver fluke infections were detected only in adult animals (after first calving or older than 2.5 years). However, some farms had not sampled enough young animals. Most authors also found that the prevalence of rumen flukes was higher in animals older than 2.5 years than in younger animals [20,21,37], while others did not show any association between age and rumen fluke infection [42]. Since the drug used for deworming was not specified for 144 farms, the effect of deworming on rumen and liver fluke prevalence could not be conclusively determined.

## 5. Conclusions

Our study revealed that the prevalence of rumen flukes is generally rather low in Germany and distributes unevenly. Comparing rumen and liver fluke occurrence, the distribution between the regions North and South is counterbalanced. *C. daubneyi* was the most frequently identified species, while *P. leydeni* was first recorded in Germany. A statistically significant association was observed between the prevalence of flukes and grazing/feeding fresh grass and, therefore, also between prevalence and organic farming and additionally, in case of rumen flukes, with suckler cow husbandry. For reasons of animal welfare, animal health and economic viability, the fluke prevalence in Germany should continue to be monitored or repeated in a few years. Perhaps a serological method for rumen flukes can than already be used [14] in addition to serological liver fluke detection. Finally, paramphistomidosis should be given more attention by veterinarians and farmers [43] and many (epidemiological) research questions remain to be addressed.

## Figures and Tables

**Table 1:** Number of sampled farms per German region and federal state.

Region	Number of Farms per Region	Federal State	Number of Farms per Federal State
North	179	Schleswig-Holstein	51
		Hamburg	1
		Lower Saxony	92
		Bremen	3
		Mecklenburg-Western Pomerania	32
East	76	Berlin	0
		Brandenburg	16
		Saxony	9
		Saxony-Anhalt	15
		Thuringia	36
South	277	Baden-Wurttemberg	72
		Bavaria	205
West	82	North Rhine-Westphalia	45
		Hesse	20
		Rhineland-Palatinate	16
		Saarland	1
Germany			614

**Table 2:** Odds ratio, 95% confidence interval and *p*-value for rumen fluke and *F. hepatica* prevalence per German region. Note that in the region West no *F. hepatica* positive samples were observed.

Predictor	OR	95% CI	*p*-Value
Rumen Flukes			
South (Intercept)	0.04	0.02–0.07	<0.001
North	2.29	1.08–5.76	0.038
East	2.10	0.75–6.41	0.145
West	0.97	0.22–3.43	0.965
*F. hepatica*			
South (Intercept)	0.17	0.12–0.24	<0.001
North	0.39	0.18–0.73	0.007
East	0.52	0.18–1.13	0.134

Abbreviations: OR, odds ratio; CI, confidence interval.

**Table 3:** Prevalence of patent rumen fluke, *F. hepatica* and co-infections in German cattle farms (*n* = 614) related to the management factors production type and agricultural system. Note that percent calculation was omitted if less than five farms (total values) were included.

	Total	Rumen Flukes ^a^		95% CI	*F. hepatica* ^a^		95% CI	Co-Infection		95% CI
	*n*	*n*	%	%	*n*	%	%	*n*	%	%
**Production type**										
Dairy cows	571	23	4.0	2.8–6.1	54	9.5	7.3–12.1	9	1.6	0.9–3.1
Suckler cows	43	11	25.6	14.2–39.4	4	9.3	3.7–21.6	4	9.3	3.2–20.8
**Agricultural system**										
Organic	106	11	10.4	5.7–17.4	29	27.4	19.5–36.2	3	2.8	0.9–7.9
Conventional	506	22	4.3	3.0–6.6	29	5.7	4.1–8.2	10	2.0	1.1–3.6
No information	2	1	n. a.	n. a.	0	n. a.	n. a.	0	n. a.	n. a.

^a^. Including coinfections. Abbreviations: n. a., not applicable; CI, confidence interval.

**Table 4:** Descriptive data on the occurrence of patent rumen fluke, *F. hepatica* and co-infections in German cattle farms (*n* = 614) related to the management factors grazing/feeding fresh grass and anthelminthic treatment. Note that percent calculation was omitted if less than five farms (total values) were included.

	Total	Negative		Rumen Flukes ^a^		*F. hepatica* ^a^		Co-Infection	
	*n*	*n*	%	*n*	%	*n*	%	*n*	%
**Grazing/feeding fresh grass**									
All herds with access	457	381	83.4	33	7.2	56	12.3	13	2.8
All herds without access	156	153	98.1	1	0.6	2	1.3	0	0.0
Dairy cow herds with access	415	350	84.3	22	5.3	52	12.5	9	2.2
Dairy cow herds without access	155	152	98.1	1	0.6	2	1.3	0	0.0
Suckler cow herds with access	42	31	73.8	11	26.2	4	9.5	4	9.5
Suckler cow herds without access	1	1	n. a.	0	n. a.	0	n. a.	0	n. a.
No information	1	1	n. a.	0	n. a.	0	n. a.	0	n. a.
**Anthelminthic treatment**									
None	338	311	92.0	8	2.4	20	5.9	1	0.3
Fasciolicides	29	20	69.0	5	17.2	5	17.2	1	3.4
Others than fasciolicides	101	85	84.2	11	10.9	11	10.9	6	5.9
Not specified	144	118	81.9	10	6.9	21	14.6	5	3.5
No information	2	1	n. a.	0	n. a.	1	n. a.	0	n. a.

^a^. Including coinfections. Abbreviations: n. a., not applicable.

## Data Availability

Data supporting reported results is contained within the article.
